# Patients' experiences and perceived causes of persisting discomfort following day surgery

**DOI:** 10.1186/1472-6955-9-16

**Published:** 2010-10-27

**Authors:** Helena I Rosén, Ingrid HE Bergh, Berit M Lundman, Lena B Mårtensson

**Affiliations:** 1 School of Life Sciences, University of Skövde, Box 408, Skövde, (SE-541 28), Sweden; 2 Institute of Health and Care Sciences, The Sahlgrenska Academy at University of Gothenburg, Box 457, Gothenburg, (SE-405 30), Sweden; 3 College of Nursing, University of Rhode Island, (2 Heathman Road), Kingston, (RI 02881-2021), USA; 4 Institute of Nursing, Umeå University, (SE-901 87), Umeå, Sweden

## Abstract

**Background:**

The aim of this study was to describe patients' experiences and perceived causes of persisting discomfort following day surgery. Earlier research has mainly covered symptoms and signs during a recovery period of up to one month, and not dealt with patients' perceptions of what causes persisting, longer-term discomfort.

**Methods:**

This study is a part from a study carried out during the period May 2006 to May 2007 with a total of 298 day surgery patients. Answers were completed by 118 patients at 48 hours, 110 at seven days and 46 at three months to one open-ended question related to discomfort after day surgery constructed as follows: *If you are still experiencing discomfort related to the surgery, what is the reason, in your opinion*? Data was processed, quantitatively and qualitatively. Descriptive, inferential, correlation and content analyses were performed.

**Results:**

The results suggest that patients suffer from remaining discomfort e.g. pain and wound problem, with effects on daily life following day surgery up to three months. Among patients' perceptions of *factors leading to discomfort *may be *wrongful or suboptimal treatment*, *type of surgery *or *insufficient access to provider/information.*

**Conclusions:**

The results have important implications for preventing and managing discomfort at home following day surgery, and for nursing interventions to help patients handle the recovery period better.

## Background

Patients' discomfort, physiological and psychological/psychosocial, e.g. pain, nausea and anxiety, during the recovery period at home following day surgery is more profound and lasts longer than was previously known. This discomfort interferes with the ability to return to normal activities such as laundry, shopping and work outside the home [1,2]. Discomfort can be related to the surgical wound [3], lack of knowledge or lack of information related to the surgery [4,5]. When people experience discomfort they interpret their experience and search for a cause. This phenomenon has been described as a personal understanding of illness. Most research on personal understanding of disease has focused on chronic diseases [6-8].

Sophisticated technologies, medical advances and modern anesthetics have resulted in shortened hospitalizations for inpatients [9,10] and a rapid expansion of day surgery [11]. Up to 70% of elective operations are currently performed as day surgery in the industrialized world [12]. As a result, responsibility for postoperative recovery is being shifted from professionals to the patients and their families. There is some knowledge of patients' ability to manage their own recovery and their experiences of the postoperative recovery process [13]. However, only a few studies have examined patients' experiences and symptoms during the recovery period following day surgery over time. When studied from postoperative day one to seven and after one month and six months, patients' perceptions of symptom occurrence and distress decreased during the first week. However, 30% of participating patients still reported at least one episode of distress or one symptom at six months [14]. Pain is the most prominent symptom after day surgery [15-18] but symptoms such as nausea, tiredness, vomiting, headache, drowsiness, dizziness, sore throat and backache are also common [18,19]. Pain, fatigue and impaired function are postoperative discomfort that can persist over a long period and that also seem to affect return to usual activities after day surgery [17].

Symptoms are subjective experiences reflecting changes in a person's biopsychosocial function, sensation or cognition. Signs are any abnormality or signs of disease that can be detected by another person or the patient himself [20]. Earlier research has mainly covered symptoms and signs during a recovery period of up to one month [1]. To our best knowledge, there are no studies of patients' perceptions of what causes persisting, longer-term discomfort. The aim of this study is to describe patients' experiences and perceived causes of persisting discomfort following day surgery.

## Methods

### Research design

A descriptive design was adopted. This paper focuses on answers to one open-ended question related to discomfort after day surgery.

### Data sample and collection

According to official national Swedish health statistics, approximately 623 000 operations were performed on adults (20-85+) as day surgery in Sweden during 2008. This represents 64% of all operations in Sweden [21]. Most hospitals have standardized routines for discharge and follow-up telephone calls. Approximately 40% of the day surgery units make follow-up telephone calls to all patients [22]. Patients were consecutively selected from a waiting list. These data are a part of a larger project carried out during the period May 2006 to May 2007 at a day surgery unit in a county hospital in Sweden in which inclusion criteria were: age 18 and above and ability to read and write Swedish. Quantitative data from the main project have been analysed and will be presented elsewhere. Answers, to one open-ended question from that project are analysed in this study. Patients who were about to undergo day surgery received information about the study in a letter that was sent out simultaneously with the notification of the scheduled operation. At admission, patients who were willing to participate in the study gave written consent, together with their address and telephone number. Due to the inclusion criteria a total of 435 were asked to participate. Of those 93 declined and 44 were excluded or withdrew, 298 remained (general surgery n = 205; urology n = 60; orthopedic n = 33). They were equally divided in terms of gender (male 49% and female 51%) and ranged in age from 18 to 88 (mean age 54). Data collection was performed by one of the authors (HR), aided by a research assistant. The open-ended question was constructed as follows: *If you are still experiencing discomfort related to the surgery, what is the reason, in your opinion? *All patients were distinctly informed that the item referred to surgery-related discomfort. The included patients in the present study underwent a wide range of surgery procedures (e.g. ligation/resection of vena saphena magna, radical surgery inguinal hernia, haemorrhoidectomy and arthroscopy).

### Procedure

After giving informed consent, the patients received an envelope with written information about the study and the questionnaires to be answered at home 48 hours and seven days following surgery. The questionnaire to be answered three months after surgery was sent by postal mail. All questionnaires were requested to be returned by mail and stamps and envelopes were provided by the researchers.

### Data analysis

Responses irrelevant to this study, such as comments on question construction and reports denying persisting discomfort or describing discomfort existing prior to the surgery, were excluded. The remaining data was processed, quantitatively and qualitatively.

### Statistics

Simple quantitative analyses of patterns and regularities were performed for the responses: 118 at 48 hours, 110 at seven days and 46 at three months. Significant differences between the group with (n = 46) and the group without (n = 252) persisting discomfort at three months were calculated by Chi square test with two degrees of freedom. Gender was analyzed by Fisher's exact test and mean age by t-test. All tests were two-tailed at the significance level p ≤ 0.05. The results were analyzed with SPSS for Windows version 14.0.

### Qualitative content analysis

In order to evaluate responses to open-ended questions in a questionnaire, qualitative content analysis has been judged as being suitable [23]. The analysis was performed on the data for those 46 patients who had remaining discomfort at three months and for those who had also reported discomfort at 48 hours and/or seven days. In this study, interpretation of content focused on what the text said, i.e. the manifest content. The analytical strategy in this study was guided by Graneheim and Lundman [24] and the units of analysis were first inserted into a table and then analyzed as follows:

1) familiarization of data, i.e. the units of content were read and re-read, 2) condensation of original statements when required: no further processing occurred before the coding process, 3) data-derived coding of all units of content related to discomfort, 4) summary of the codes into sub-categories, 5) summary of the sub-categories into categories, 6) simultaneous and continuous modification of codes, sub-categories and categories during the course of the analysis, 7) organization of categories into major topics, 8) summary and presentation of sub-categories and categories.

### Ethics

The ethical perspectives and rules in the Helsinki declaration were observed [25]. Written informed consent was obtained from each patient. The invitation letter sent to the patients emphasized confidentiality and the fact that withdrawal was possible at any time without giving a reason and would not affect their current or future care in any way. The study was approved by the Ethics Committee at The University of Gothenburg, Sweden (approval nr. 030-06).

## Results

In order to clarify the outcome, the patients were divided into groups: the 118 respondents at 48 hours are referred to as Group 1, and the 110 at 7 days and the 46 at 3 months are referred to as Groups 2 and 3, respectively.

### Frequency of discomfort in Groups 1 (48 hours), 2 (7 days) and 3 (3 months)

The respective frequencies of discomfort in all three groups are presented in Table 1. While there were major variations in the types of discomfort over time, the most common type described was pain at all three time-points. Discomfort from the surgical wound was reported both at 48 hours and at seven days but the frequency decreased quite sharply over time. Bleeding or residual blood in the wound was reported at all three time-points, with a slight increase at seven days. Pneumonia was also mentioned at 48 hours. Patients who experienced discomfort at three months had also done so earlier in the recovery period, as a rule.

**Table 1:** Frequency of discomfort type at 48 hours, 7 days and 3 months, respectively, following day surgery.

	Frequency	Frequency	Frequency
Types of discomfort	***48 hours (n = 118)***	***7 days (n = 110)***	***3 month (n = 46)***
Pain^#1^	53	42	15
Wound problems^#2^	22	25	6
Swelling^#3^	9	16	2
Urinary tract problems^#4^	6	4	3
Bleeding^#5^	5	9	6
Headache	4		
Stiffness^#6^	4	3	1
Nausea	3	2	2
Dizziness	2		
Anxiety	1	1	
Depression	1		
Exhaustion	1		
Poor appetite	1		
Spasms	1	1	
Drowsiness		1	
Itching			1
Numbness			1
Defecation problems		1	
Sore throat		1	

Patients may have reported more than one symptom at each time-point. Orderly by the frequency at 48 hours.

#1 Pain characteristics e.g. dull or pulsating, location e.g. groin or rectum, situations e.g. contact or when moving

# 2 E.g. tender or pus filled

#3 E.g. bruises or undefined

#4 E.g. difficulty emptying bladder or frequent urge to urinate

#5 E.g. fresh or residual blood from the surgery area

#6 E.g. stiffness in wound or body parts undergoing surgery

### Discomfort over time in Group 3 (3 months)

When the 46 patients with persisting discomfort after 3 months were compared with the 252 not reporting any persisting discomfort, the former were significantly older than the latter (mean 58 vs 53 years, p = 0.0175, t-test) but there was no significant difference in gender (p = 0.1282, Fisher's exact test). The majority (n = 34) of those with persisting discomfort had undergone their procedures at a general surgery clinic while six were orthopedic surgery patients and six were urology patients. The groups with and without persisting discomfort did not differ in terms of surgical specialty (p = 0.7756, Chi square test with two degrees of freedom).

The experiences and perceived causes of persisting discomfort following day surgery varied in Group 3 see Table 2. A majority in this group had experienced discomfort at 48 hours (n = 31) and at seven days (n = 37) after surgery as well. The categories that emerged in Group 3 are presented in Table 2. The subcategories indicate variability in patients' understanding of what caused their persisting discomfort.

**Table 2:** Categories emerging over time for the 46 patients who experienced persisting discomforts three months after day surgery.

Categories	Sub-categories		
	***48 hours (n = 31)***	***7 days (n = 37)***	***3 months (n = 46)***
**Discomfort**	Anxiety	Anxiety	
	Bleeding	Bleeding	Bleeding
	Dizziness		
	Headache		
			Itching
	Nausea		Nausea
	Pain	Pain	Pain
	Sadness		
	Stiffness		Stiffness
	Spasms	Spasms	
	Swelling	Swelling	Swelling
	Tiredness		
			Urinary tract problems
	Wound problems	Wound problems	Wound problems
			
**Factors leading to discomfort**	Type of surgery	Type of surgery	Type of surgery
		Insufficient access to provider/information	Insufficient access to provider/information
	Incorrect or suboptimal treatment	Incorrect or suboptimal treatment	Incorrect or suboptimal treatment

Alphabetical order.

Table 2 presents the types of discomfort and certain factors leading to discomfort, according to the patients' perceptions. Varying types of discomfort were reported over time: each time-point in Group 3 will thus be illustrated with quotes below. Categories and subcategories are written in bold italics.

### Types of discomfort in group 1 (48 hours)

Discomfort was described as a sign indicating a complication, e.g. ***bleeding: **Bleeding after the first surgery which necessitated two further surgical procedures*. It was also reported from the urethra and anus after surgery in those areas in other cases. Side effects such as ***dizziness ***and ***headache ***were described at this time-point: *I got a packet of pain medication to take home... I was supposed to take two Citodon (paracetamol-codeine) every 6^th ^hour... I took it three times but I felt terrible......nausea, dizziness and headache. **Nausea ***was explained as due to the surgery itself or, for example, ***anxiety***: *After the surgery I felt nauseous...nausea because of the tension and anxiety*.

***Pain ***was connected to the surgical wound that was sore and swollen in some cases, and impacted on daily function such as the ability to sleep. *I couldn't sleep the first night because of the pain, but the second night I did manage to sleep a couple of hours at a time*. Patients also experienced ***tiredness ***and described exhaustion interfering with daily function: *have also been very tired, have no energy whatsoever. Get dizzy easily. *Patients also described thoughts and feelings such as ***sadness***: ...*have been sad and depressed since the surgery. I don't know why. *Some experienced postoperative ***stiffness ***in various locations, such as the wound or the body part in which the surgery was performed: *A little stiff and sore after surgery in my groin and thigh. **Spasms ***in the anal sphincter was another type of discomfort, one patient who had his hemorrhoids removed described this as: *Spasms for about ten minutes after voiding and then I felt OK...**Swelling ***was described as a consequence of the healing process or of bleeding in the surgery area: *The day after surgery, I was really swollen... turned out to be a major hemorrhage*. Patients described ***tiredness ***as being caused by the fact that they could not always sleep in their accustomed position or because of tension due to pain. ***Wound problems ***at 48 hours tended to be regarded as a natural process, apparently deriving from the understanding that time heals wounds: discomfort was interpreted as being due to the fact that the healing process was still ongoing: *I guess the pain is natural and that it will get better when the wound is healed.*

### Types of discomfort in group 2 (7 days)

Some patients expressed ***anxiety ***at having been too active: *It might be because you get so much pain medication...since you don't feel so much you might move around too much. *Varying types of discomfort, such as blood accumulation in the surgical wound and big bruises on the leg after radical inguinal hernia, occurred at seven days: *Since the wound bled after the surgery and it kept on **bleeding **that night, I think blood might have accumulated inside it, because I've got burning and smarting pain and my belly and down toward my groin are numb.*. Patients also described ***pain ***in different situations. *It hurts when I stand still and after having sat with my legs up, and it hurts when I start to walk again*. Pain types reported were soreness, smarting and burning pain: *It's sore after surgery.... burning and smarting pain. *Like at 48 hours, discomfort in the sphincter was described as: ***spasms **for about ten minutes after voiding. **Swelling ***in the surgical scar was also mentioned: bleeding inside the wound was a perceived cause. ***Wound problems, ***such as swelling, were experienced as worse when the individual was moving. Inflammation of the wound also occurred. At seven days patients also stated that the healing process was ongoing: *The healing process isn't finished *and ...*tender and swollen wound. It hasn't healed*. At both 48 hours and seven days following surgery, swelling and tenderness were considered to be normal reactions after surgery.

### Types of discomfort in group 3 (3 months)

***Bleeding ***was considered to be due to old accumulated blood: ...it *began to bleed profusely from a surgical incision. Was old blood that remained... *or to be surface bleeding after hemorrhoidectomy when blood was seen on toilet paper. ***Nausea ***was explained as caused by special kinds of drink or food, connected with abdominal pain, or else the cause was unclear: *That nausea sometimes comes in the evenings, not really sure why but it's probably nothing to worry about. *In addition to the surgery and to being in motion, patients also connected ***pain ***with the surgical wound, urinating or defecating: *The wound does not heal. I have still pain and bleeding. *Types of pain experienced were pressure and soreness. Patients also experienced discomfort from the ***urinary tract***. For example, some described a frequent need to urinate when sitting in certain positions, which disturbed their activities. *... that I have frequent urges to pee and have to pee often and when I lean back, for example in a sofa, I get the urge and it's really difficult because the more often I go the worse it gets. **Itching ***and blood on toilet paper were described by patients as being due to hemorroidectomy: ...*itching and cracking after a bowel movement*. The experiences of discomfort proved to differ to a great extent. After hand surgery one patient had still had ***numbness ***in the thumb: *The pain is gone but I have not got back all the feeling in my thumb yet*. A cosmetically unappealing scar was experienced as discomfort by another. Patients also described abnormalities indicating disease. One had problems with urinary incontinence that had been cured but returned and another thought that the signs were likely due to inflammation after varicose vein surgery: *It's probably an inflammation - it comes from the groin where they pulled the blood vessel out. The **swelling **and **stiffness **started about 4 1/2 weeks after the surgery*.

Patients with remaining ***wound problems ***at three months referred to the long time it was taking for the wound to heal and did not always perceive a reason for it: *The wound isn't healing......it still hurts and is bleeding......cause??? *There were also descriptions of swollen, red and pus-filled wounds.

### Factors leading to discomfort in Group 3 (3 months)

In the following, the patients' perceived causes of persisting discomfort will be presented. The descriptions seem to be linear, but they contain an intermingling of different symptoms and perceived causes. At seven days and three months insufficient access to health care and information were experienced and perceived as factors leading to discomfort. This discomfort was described as physiological as well as in psychological/psychosocial terms.

The patients believed that something had gone wrong with the surgery, i.e. ***incorrect or suboptimal treatment***. Some also believed that it was the very nature of the surgery, i.e. the ***type of surgery***, they had undergone, that caused the discomfort. Furthermore, some patients expressed frustration with encounters with health care professionals, the results of the surgery, or ***insufficient access ***to care or ***information***, and some expressed a need for treatment e.g. wound treatment.

***Type of surgery ***was a perceived cause of discomfort at all three time-points *I will never have haemorrhoid surgery again. It was dreadful. *At the first two time-points, some had reported, briefly, that they believed that something was amiss, i.e. ***incorrect or suboptimal treatment***: *Something went wrong or else I wasn't reacting normally after surgery*. At three months they elaborated, expressing the conviction that carelessness or lack of skill on the part of the surgeon had caused their discomfort: *I think my surgery went wrong. The doctor who operated on the patient in the next bed said, ' Stand up so I can make sure I've got the right one.', but my doctor only read his piece of paper and that's why it turned out like it did*. Some patients also regarded repeat surgery as a cause of discomfort. Some underwent repeat surgery on account of discovered pathology for example, a renal calculus was found when an x-ray was done during the first operation or not enough tissue being removed the first time. Patients who considered ***incorrect or suboptimal treatment ***to be the cause of persisting discomfort expressed feelings of anger, disappointment and sadness. Strong feelings of anger at the surgeon emerged because of his/her bedside manner or because the results were unsatisfactory: *Everything sucks, I'm being treated like shit ... my leg looks terrible now, even worse than before*. In the subcategory ***incorrect or suboptimal treatment ***there were also aspects associated with feeling of distrust in the providers and, indirectly, in the structure of the organization: *Poor advice and help. Then received a notice to attend which I thought was a return visit. It was a referral which I would have had to come along for before the operation. I just had to get on with it and go home. On top of that was the 3-month waiting period. Maybe you should review these procedures.*

Patients reported **insufficient access**, both to contact with health care professionals and to **information**, at 48 hours and seven days: *I contacted the nurse at the surgery department and she said she would talk to the physician who operated on me. He's off duty this week so they'll let me know later on when my follow-up appointment will be scheduled*.

Patients experienced that complications occurred while they were waiting to get in touch with the health care system. For example, a wound ruptured, releasing pus and serum, the day after a patient had tried to come into contact with the surgeon for advice. Patients also reported unsatisfactory information when they had problems related to the surgery and encounters with healthcare professionals were inadequate: *I was disappointed when I called the day surgery ward after just over a week; the nurse said everything was normal even though I told her about the swelling in my groin, belly and private parts. The whole area is pulsating. I suspect an infection. The nurse said that it is normal even though it aches really badly, I called the nurse - she thought it looked all right and agreed to ask the doctor to call me up, I called the emergency room according to the instructions I was given. They weren't willing to help me at all. This period was VERY tough. Bad information and help.*

Several of the patients with persisting discomfort expressed a need for health care but they were left to rely on their own advice and care: *They weren't willing to help me at all. On my own initiative, I started using aluminum acetotartrate dressings which I changed often. I've started to treat it with anti-inflammatory gel and I think it's better already*. However, some actually were treated, at the primary care center, at the hospital's surgery clinic, at the emergency room or by admission to hospital for a few days. Others were waiting to be examined or had been given prescriptions for medication.

### Impact on daily function in groups 1 (48 hours), 2 (7 days) and 3 (3 months)

All groups reported the discomfort's impact on daily living at all three time-points, e.g. being unable to live normally, problems walking, feeling inhibited at the idea of having sex, restricted mobility or impact on sleep: *I can't walk as much as I want to. *Tiredness in the leg when walking, standing or exercising was another example of impact on normal function: *My leg gets really tired and the varicose vein pulsates when I exert myself and work out*.

## Discussion

The main finding in this study is the occurrence of discomfort, e.g. pain and wound problems and their impact on daily living, up to three months following day surgery. This implies that the recovery period is extended over a longer period than was previously known. The patients' perceptions of what caused their persisting discomfort included type of surgery, incorrect treatment and insufficient access to health care providers and information. Hence, the patients expressed that they were left to rely on their own advice/care due to insufficient access to health care and/or information or to incorrect or suboptimal treatment.

Pain and wound problems such as bleeding and swelling were the most prominent symptoms that the patients described consistently during the follow-up period. Recent studies suggest that pain is aggravated after discharge following day surgery in the majority of patients [18]. Surprisingly, six patients reported bleeding from the surgical area as late as after three months. This implies that postoperative signs and symptoms tend to persist for a longer period than physicians expect [13,26].

Varying types of discomfort, such as bleeding, pain and swelling, were reported at up to three months after surgery. Those types of discomfort were reported to have an impact on daily living, e.g. inability to live normally, problems walking, feeling inhibited at the idea of having sex, restricted mobility or impact on sleep. Patients have a tendency to assess discomfort from the perspective of its consequences for daily life [27], while clinicians often have a more scientific attitude and expect patients to follow their professional advice [28]. It is thus important, in order to increase compliance regarding e.g. use of analgesics, to ask the patient his/her opinion of the cause of the discomfort, resulting in knowledge that will facilitate mutual decision-making with patients and increase adherence to management and advice, leading to improved well-being.

Research on the impact of discomfort and symptoms on daily function usually covers the postoperative period up to one week [18]. However, we found that discomfort and symptoms could impact the patient's daily life as long as three months after the surgery. The experience of the recovery period may be dependent on appropriate and repeated information and the possibility to maintain contact with health care providers [13]. The patients in this study were dissatisfied with the information they had received concerning what to expect and how to treat various types of discomfort and symptoms during the recovery period. This is a bit surprising in Sweden, where most hospitals (96%) offer written information on the procedure, written and verbal information about pain management (100%) and a telephone number for further information (96%) [22]. Furthermore, all patients in this study were offered a follow-up call the morning after surgery, in contrast to most other hospitals in Sweden (31 of 78 units) [22]. The issue of the most appropriate time-point(s) for this follow-up call remains to be resolved. However, it has been suggested that 12-24 hours after surgery may be the most helpful time for patients [29]. Horvath [30] found that patients reported moderate to severe pain in the afternoon on postoperative day one and that pain ratings on postoperative day two were the most significant predictor of late return to usual activities. Our results at 48 hours are in agreement with Horvath. However, in our study, pain was widespread at all three studied time-points and the fact that bleeding and swelling were mentioned even more often at seven days than at 48 hours indicates a need for extended contact with health care providers.

Several of the patients were convinced that the surgery had been incorrectly performed and/or had experienced unsatisfactory encounters with health care professionals. This indicates that something went wrong in the communication between the patient and the health care professionals, which is serious and indicates a need for enhanced professional information. As patients are now in charge of preoperative preparation and recovery, information provision is a challenge for day surgery. The timing of information provision needs to be coordinated since patients will receive information at different stages and from different healthcare professionals. In addition, personalised information is beneficial to patients [4,5,31].

This finding indicates a need for interventions, including improved possibilities to obtain help and professional information. The type of surgery may not be of decisive importance for the duration of the recovery period and, consequently, for return to work following surgery. Individual recovery, influenced by diverse factors, makes it difficult to generalize when patients will be able to return to normal activity, including work [13,26].

Guidelines from day surgery units in Sweden recommend that both written and verbal information be provided in connection with discharge. Furthermore, most patients are also offered a follow-up call after surgery [22]. Despite this, the patients in this study perceived that they had had insufficient access to health care and/or information, had undergone incorrect or suboptimal treatment or were left to rely on their own advice/care. For some patients in this study, this discomfort persisted as long as up to three months after surgery. This implies that guidelines and information to patients must include an individual aspect and that it is important to help patients with their concerns over their progress. It is difficult to ascertain, or create guidelines stipulating, the expected duration of recovery periods after various types of surgical procedures. Factors unrelated to the surgery type, such as the individual level of dependence in daily life and other contextual factors, must be taken into consideration, in addition to the standard length of the recovery period on the group level [32]. For example, Integrated Care Pathways (ICP) is described by Rivas [33] as the operational version of guidelines, defining when, how and in which sequence attention and care must be given, and also specifying the objectives of each phase. Follow-up care planned in extended ICP, possibly in cooperation with community health nurses [34] may help to identify the need for care in patients that have undergone day surgery.

## Limitations and strengths

One strength of this study is that the results are based on the patients' own descriptions of their problems over time. The advantage of the descriptive results is that they can serve as a basis for further research. They can be used as the basis for purely quantitative, experimental or qualitative, interview studies. The internal attrition rate for the open-ended question, especially at three months, is a limitation. The fact that the three-month questionnaire was sent by mail may have affected the response rate negatively, because personal contact with respondents yields a high response rate [35]. The response rate may also have been affected by patients being tired of answering the questionnaires; they might have been especially unmotivated if they were no longer suffering any discomfort related to the surgery. Perhaps not everyone that experienced discomfort at 48 hours and 7 days reported this, possibly due to the hope that it would disappear. It is also possible that discomfort came and went between time-points. The trustworthiness of this study is based on a number of criteria such as the population, consisting of 298 patients of various ages having undergone various surgery types. However, the experiences of the patients who did not answer the open-ended question remain unknown. Furthermore, our results are not based on a generalizable number of patients. However, the overall high response rate in this study indicates that the results can be transferred to patients undergoing day surgery in Sweden, but it is uncertain to what extent they can be transferred to other cultures and/or healthcare systems.

## Conclusions and clinical implications

Discomfort during the recovery period after day surgery was experienced in a varying and deeply personal way and extended over a longer period than has previously been known. Patients with remaining discomfort at three months or more had also generally suffered discomfort, and its respective effects on daily life, earlier in the recovery period as well. Pain was the most common type of discomfort described. Among patients' perceptions of the factors leading to such discomfort are incorrect or suboptimal treatment, type of surgery and insufficient access to health care providers or appropriate information when seeking help for persisting discomfort. Some patients in this study stated their conviction that a mistake had been made during the operation or their belief that it was the very nature of the surgery that was the cause of the discomfort. These findings implies the importance of asking the patient what he/she believes is the cause of the discomfort in order to facilitate mutual decision-making and increase adherence to management and advice, leading to improved well-being. Nursing interventions, to create management strategies for patients experiencing persisting discomfort may be needed. In addition, further research is necessary to find out which patients are at risk of experiencing discomfort over a longer period after day surgery.

## Competing interests

The authors declare that they have no competing interests.

## Authors' contributions

HR, LM and BL were responsible for the study conception and design. HR was responsible for and undertook part of the data collection. HR, LM, IB and BL were responsible for data analysis. HR, LM, IB and BL were responsible for drafting the manuscript. HR, LM, BL and IB made critical revisions of the paper. All authors read and approved the final manuscript.

## Pre-publication history

The pre-publication history for this paper can be accessed here:

http://www.biomedcentral.com/1472-6955/9/16/prepub
